# Evaluation of layered tissue scattering properties: a time-domain spatially resolved spectroscopy approach

**DOI:** 10.1117/1.JBO.30.7.075002

**Published:** 2025-07-21

**Authors:** Elisabetta Avanzi, Laura Di Sieno, Alberto Dalla Mora, Lorenzo Spinelli, Alessandro Torricelli

**Affiliations:** a Politecnico di Milano, Dipartimento di Fisica, Milan, Italy; b Istituto di Fotonica e Nanotecnologie, Consiglio Nazionale delle Ricerche, Milan, Italy

**Keywords:** time domain, spatially resolved spectroscopy, reduced scattering coefficient, two-layer diffusive medium

## Abstract

**Significance:**

The spatially resolved spectroscopy (SRS) approach is widely used in continuous wave near-infrared spectroscopy to estimate tissue oxygen saturation in the skeletal muscle and cerebral cortex. The extension of the SRS approach to the time domain (TD) has never been proposed. We hypothesize that the time-domain spatially resolved spectroscopy (TD SRS) approach, relying on simple models and linear fit, avoiding nonlinear model-based analysis approaches, could be able to assess the homogeneity of the scattering of the explored tissue.

**Aim:**

We aim to explore the potential of the TD SRS approach for estimating $\mu_{s}^{\prime }$ from the spatial derivative of the measured signal in a homogeneous and in a two-layer medium and by considering also the effect of the instrument response function (IRF).

**Approach:**

A theoretical expression for $\mu_{s}^{\prime }$ depending on the spatial derivative of the attenuation is derived. Then, numerical simulations are conducted using solutions of the radiative transfer equation under the diffusion approximation. We consider a reflectance geometry with source–detector distance in the range 1 to 5 cm in 0.5 cm step, either in a homogenous semi-infinite or two-layer diffusive medium. Convolution with a real IRF is also carried out to mimic experimental scenarios.

**Results:**

In a homogeneous medium, the TD SRS approach is able to retrieve $\mu_{s}^{\prime }$ over a large range of values, being minimally affected by the IRF. In a two-layer medium, the TD SRS approach can only provide information on the changes of $\mu_{s}^{\prime }$ with depth but fails to provide a robust estimate of the absolute value of $\mu_{s}^{\prime }$ in either of the two layers. Moreover, the IRF can greatly affect the results in the case of the two-layer medium.

**Conclusions:**

The TD SRS approach can be a simple way to estimate spatial changes of $\mu_{s}^{\prime }$ but not the absolute value of $\mu_{s}^{\prime }$. Care should be taken to use a TD system with proper IRF.

## 1 Introduction

Since its introduction in 1999,^1^^,^^2^ the spatially resolved spectroscopy (SRS) approach has been extensively implemented in commercial continuous wave (CW) near-infrared spectroscopy (NIRS) devices for estimating tissue oximetry in skeletal muscle or cerebral cortex.^3^ The characteristic feature of CW SRS NIRS is in fact the possibility to noninvasively provide an estimate for tissue oxygen saturation $S_{t}O_{2}$—the so-called tissue oxygenation index, or tissue saturation index—based on the spatial gradient of light attenuation detected at two wavelengths by two or three narrowly spaced detectors. The SRS approach requires the *a priori* knowledge of the spectral dependence of the reduced scattering coefficient $\mu_{s}^{\prime }(\lambda )$, that is typically approximated by a simple linear dependence $\mu_{s}^{\prime }(\lambda )\approx k(1-h\lambda )$, where k and h are empirical parameters, and λ is the wavelength. The practical implementation of CW SRS NIRS brings to a simpler and cheaper device than frequency domain NIRS or time domain (TD) NIRS that can naturally provide $S_{t}O_{2}$ thanks to the estimate of the absorption coefficient $\mu_{a}$.

In a recent work, we have studied the robustness of the CW SRS approach.^4^ Specifically, we have investigated the errors in retrieving hemodynamic parameters, in particular $S_{t}O_{2}$, when $\mu_{s}^{\prime }(\lambda )$ was varied from expected values, and when layered tissue was considered. In this work, we present the extension of the SRS approach to the TD regime.

The idea of applying the SRS methodology to the TD regime may appear a bit curious or useless. On the one side, as already mentioned, TD NIRS can simultaneously provide estimates for $\mu_{s}^{\prime }(\lambda )$ and $\mu_{a}(\lambda )$ with a single source–detector distance ρ.^5^ In addition, theoretical studies have shown that in the TD regime, the penetration depth is independent of ρ in homogeneous and layered media.^6^^,^^7^ Nonetheless, the dependence of the TD NIRS signal from ρ has been exploited to derive estimates for $\mu_{s}^{\prime }$ in a homogeneous medium without resorting to nonlinear fitting procedures.^8^ Furthermore, the use of multiple ρ in TD NIRS can be useful to improve estimates of optical properties in a layered medium^9^^,^^10^ and also to minimize the effect of a broad instrument response function (IRF) because a longer ρ naturally selects photons with longer (i.e., deeper) pathlengths.^11^ In particular, when dealing with a layered medium, the estimate of $\mu_{s}^{\prime }$ of the deeper medium is always less accurate than the estimate for the superficial layer,^9^ and a common simplistic assumption is to consider $\mu_{s}^{\prime }$ uniform in the medium.

On the basis of the above considerations, this study aims to explore the potential of the time-domain spatially resolved spectroscopy (TD SRS) approach for estimating $\mu_{s}^{\prime }$ from the spatial derivative of the measured signal in a homogeneous and in a two-layer medium and by considering also the effect of different IRFs.

## 2 Theory

We model the tissue as a semi-infinite medium with absorption coefficient $\mu_{a}$ and reduced scattering coefficient $\mu_{s}^{\prime }$, and we adopt a reflectance geometry configuration with a collimated light source and a source–detector distance ρ. Under the partial current boundary condition, the TD reflectance (the number of photons per unit area and unit time exiting the medium) at a distance ρ is given by

$$R(\rho ,t)=\frac{\Phi (\rho ,z=0,t)}{2B}=\frac{(4\pi Dt{)}^{-3/2}v^{-1/2}}{2B}[e^{\left(-\frac{z_{s}^{2}}{4Dvt}\right)}+e^{\left(-\frac{z_{b}^{2}}{4Dvt}\right)}]e^{\left(-\frac{\rho^{2}}{4Dvt}-\mu_{a}vt\right)},$$ (1)

where Φ(ρ,z,t) is the fluence, B is a parameter accounting for refractive index mismatch at the medium boundary, $D=(3\mu_{s}^{\prime }{)}^{-1}$ is the diffusion coefficient, and $z_{s}=1/\mu_{s}^{\prime }$, $z_{b}=2z_{e}+z_{s}$, $z_{e}=2BD$ is the extrapolated boundary.^12^

The TD light attenuation is defined as A(ρ,t)=−ln[R(ρ,t)]. Similar to the CW SRS approach, we calculate ∂A/∂ρ, the spatial derivative of A, as follows:

$$\frac{\partial A(\rho ,t)}{\partial \rho }=-\frac{1}{R(\rho ,t)}\frac{\partial R(\rho ,t)}{\partial \rho }=\frac{\rho }{2Dvt}=\frac{3\mu_{s}^{\prime }\rho }{2vt}.$$ (2)

From Eq. (2), we derive the expression for the reduced scattering coefficient $\mu_{s}^{\prime }$

$$\mu_{s}^{\prime }=\frac{2vt}{3\rho }\frac{\partial A(\rho ,t)}{\partial \rho }.$$ (3)

## 3 Material and Methods

### 3.1 Simulations

**Fig. 1:**
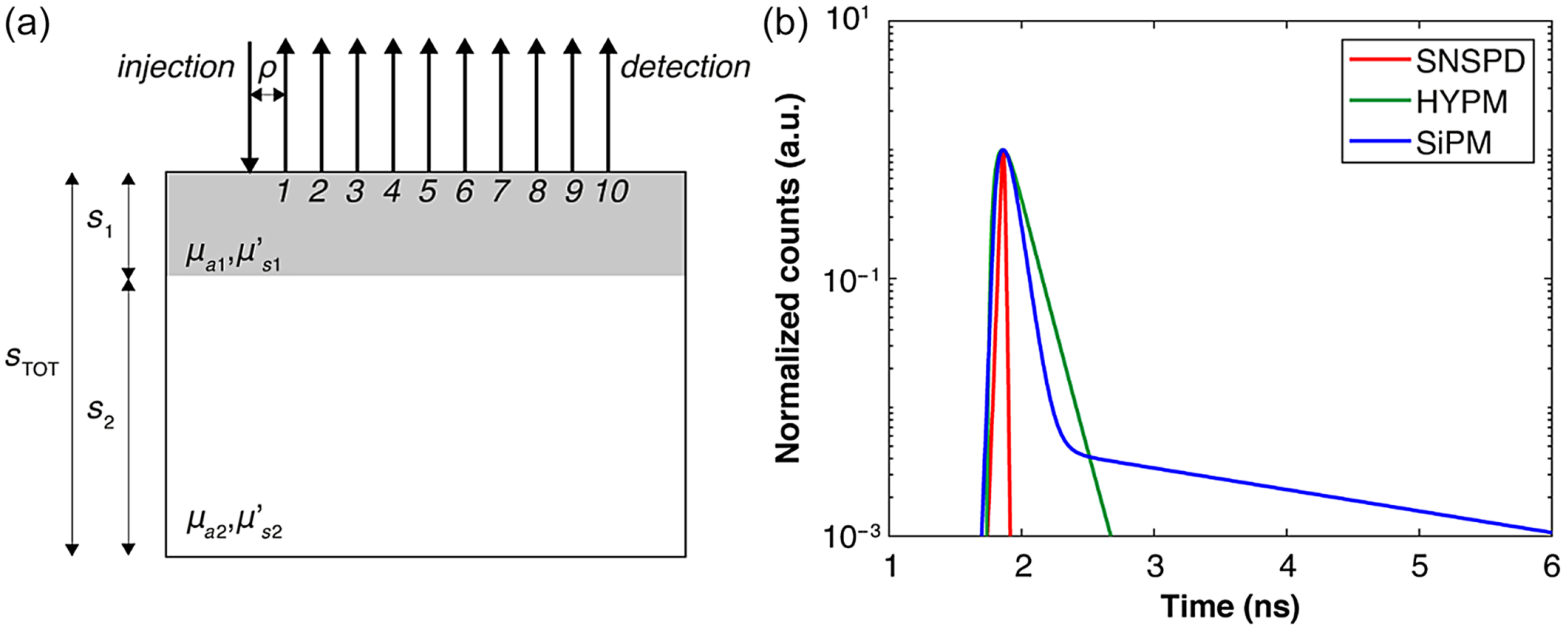
(a) Geometry used for the simulation of the two-layer structure. (b) The IRFs used for the simulation of real system data (SNSPD, superconducting nanowire single photon detector; HYPM, hybrid photomultiplier; SiPM, silicon photomultiplier).

Simulations were conducted using solutions of the radiative transfer equation under the diffusion approximation. We considered a reflectance geometry in either a homogenous semi-infinite or two-layer diffusive medium, with source–detector distances ρ ranging from 0.5 to 5 cm in 0.5 cm steps, as shown in Fig. 1(a). We set $10^{6}$ as the total number of detected photons at ρ=5  cm. The number of detected photons was properly scaled at the other source–detector distances according to the diffusion model by normalizing the area under the TD reflectance curve.

Both ideal and realistic systems were considered. In modeling the ideal system, a Dirac δ IRF was used. For the realistic system, however, the TD reflectance curves were convolved with IRFs representative of different TD systems, thereby accounting for nonideal effects such as finite duration and exponential tail. Specifically, three typical systems were examined employing, respectively, a superconducting nanowire single photon detector (SNSPD),^13^ hybrid photomultiplier (HYPM),^14^ and silicon photomultiplier (SiPM) detectors^15^ [see Fig. 1(b)]. As common simulation parameters, we used a time bin width of 5  ps/ch, a diffusive medium with a refractive index of 1.40, and a total time scale of 10 ns.

In the simulations for the homogeneous semi-infinite medium, we modeled five values of the reduced scattering coefficient ($\mu_{s1}^{\prime }=\mu_{s2}^{\prime }=5$, 7.5, 10, 12.5, and $15\;{\mathrm{cm}}^{-1}$), each paired with four absorption coefficient values ($\mu_{a1}=\mu_{a2}=0.05$, 0.1, 0.15, and $0.20\;{\mathrm{cm}}^{-1}$).

For the two-layer medium, instead, we fixed the absorption coefficient at $\mu_{a1}=\mu_{a2}=0.15\;{\mathrm{cm}}^{-1}$ and explored combinations of reduced scattering coefficients, ensuring distinct scattering properties across layers ($\mu_{s1}^{\prime }=5$, 10, $15\;{\mathrm{cm}}^{-1}$, $\mu_{s2}^{\prime }=5$, 10, $15\;{\mathrm{cm}}^{-1}$), as shown in Table 1. The overall thickness $s_{\mathrm{TOT}}$ was fixed at 9 cm, and three different thicknesses of the first layer ($s_{1}=0.5$, 1, and 1.5 cm) have been used. In S1 in the Supplementary Material, we have also studied other cases with smaller differences between $\mu_{s1}^{\prime }$ and $\mu_{s2}^{\prime }$.

**Table 1:** Various combinations of reduced scattering coefficient in the two-layer medium.

Case	$\mu_{s1}^{\prime }$ (${\mathrm{cm}}^{-1}$)	$\mu_{s2}^{\prime }$ (${\mathrm{cm}}^{-1}$)
#1	5	10
#2	5	15
#3	10	5
#4	10	15
#5	15	5
#6	15	10

### 3.2 Data Analysis

From the simulated TD reflectance R(ρ,t), we derived the spatial derivative of the attenuation A(ρ,t) at different source–detector distances $\rho_{L}$ by means of central finite differences as $\frac{\partial A(\rho_{L},t)}{\partial \rho }≅\frac{A(\rho_{L},t)-A(\rho_{S},t)}{\rho_{L}-\rho_{S}}$, with $\rho_{L}=\rho_{S}+d$, with $d\ll \rho_{S}$. As clearly described by Ref. 16, in the practical implementation when using two source–detector distances, the numerical approximation $\frac{1}{\rho }≅\frac{1}{\rho_{S}}≅\frac{\ln(\frac{\rho_{L}}{\rho_{S}})}{\rho_{L}-\rho_{S}}$ can be used in Eq. (3), yielding the following approximate expression:

$$\mu_{s}^{\prime }≅\frac{2vt}{3}\frac{\ln(\frac{\rho_{L}}{\rho_{S}})}{\rho_{L}-\rho_{S}}\frac{A(\rho_{L},t)-A(\rho_{S},t)}{\rho_{L}-\rho_{S}}.$$ (4)

We repeated the calculation of $\frac{\partial A(\rho_{L},t)}{\partial \rho }$ for $\rho_{L}$ ranging from 1 to 5 cm and using d=0.5  cm.

### 3.3 Figures of Merit

To determine whether our system can retrieve the reduced scattering coefficient with the proposed method, we evaluated two metrics based on the sample geometry. For the homogeneous geometry, we calculated the temporal average value of the retrieved reduced scattering coefficient across various media, focusing on a time window spanning from 90% of the TD reflectance curve peak on the rising edge to 10% of the peak on the tail. For the two-layer sample, we evaluated the slope of a linear fit to the retrieved reduced scattering coefficient as a function of time within a 500 ps time window starting 1 ns after the IRF peak position of each simulation condition. In the latter case, indeed, we have two values of the reduced scattering coefficient, $\mu_{s1}^{\prime }$ and $\mu_{s2}^{\prime }$, and only one retrieved value $\mu_{s}^{\prime }$. Then, the single retrieved value will depend on both $\mu_{s1}^{\prime }$ and $\mu_{s2}^{\prime }$. Because the penetration depth of photons is linked to their time-of-flight, the slope of function $\mu_{s}^{\prime }(t)$ likely brings information on the relative values of $\mu_{s1}^{\prime }$ and $\mu_{s2}^{\prime }$. Finally, we observe that the choice of a 500 ps wide temporal interval is based on our experience with TD NIRS data. This width in fact is on one side sufficiently wide to contain enough photons, avoiding noisy results, and on the other side is sufficiently narrow to maintain a reasonable temporal resolution. Moreover, the choice to start analysis at ∼1  ns after the IRF peak guarantees that we are considering portions of time-resolved curves that have enough photons, at least in the majority of cases, and also ensures that the examined slope can be confidently associated with changes in the reduced scattering coefficient.

## 4 Results

### 4.1 Homogeneous Medium

#### 4.1.1 Ideal IRF (Dirac δ)

**Fig. 2:**
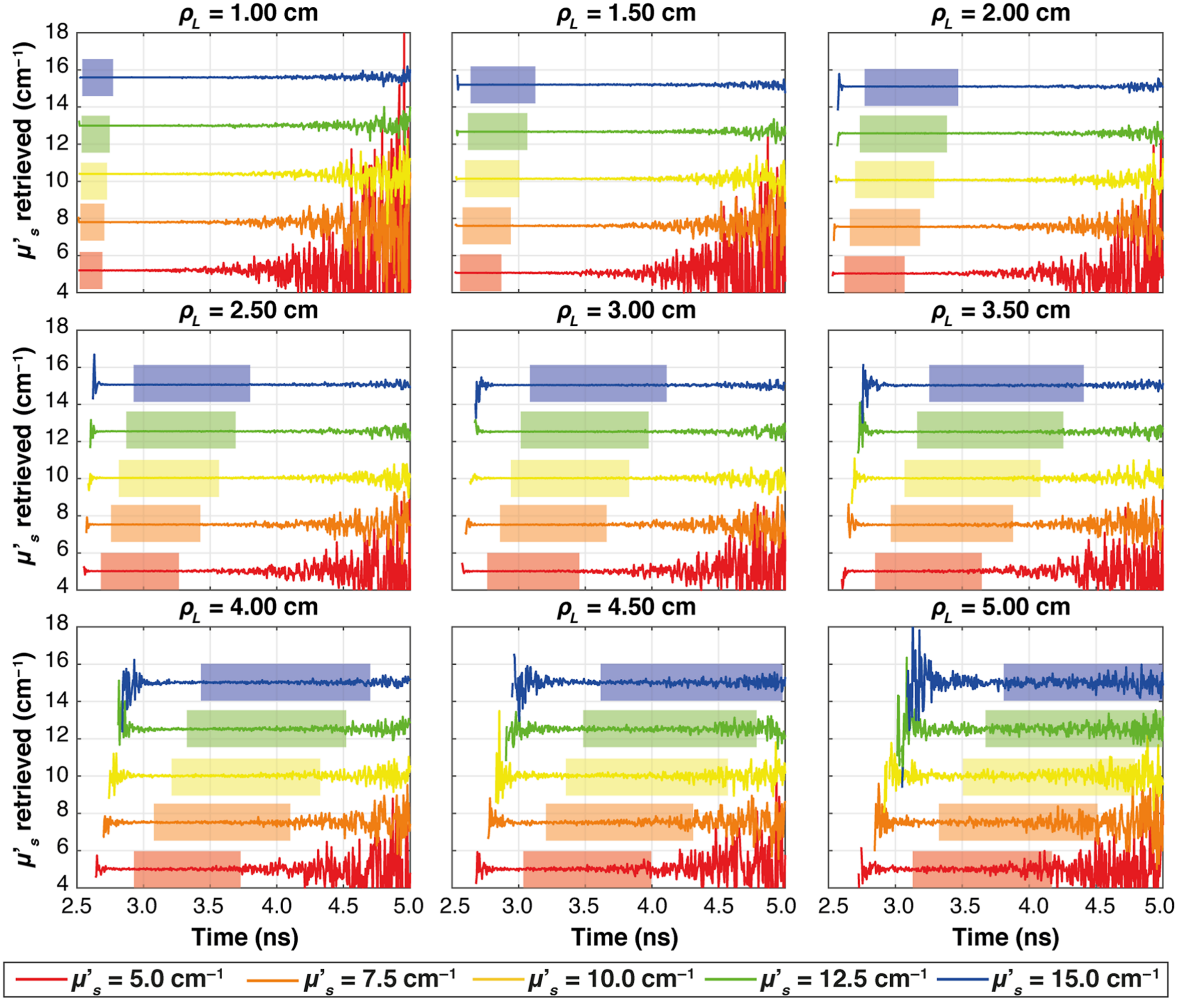
Plots of the retrieved $\mu_{s}^{\prime }$ as a function of time for all the $\rho_{L}$ at all the nominal reduced scattering coefficients (colors) in a homogeneous medium with Dirac δ IRF. The $\mu_{a}$ is set to $0.15\;{\mathrm{cm}}^{-1}$. The shaded areas extend from 90% to 10% of the peak of the TD reflectance curve and represent the time intervals used for the calculation of the average.

**Fig. 3:**
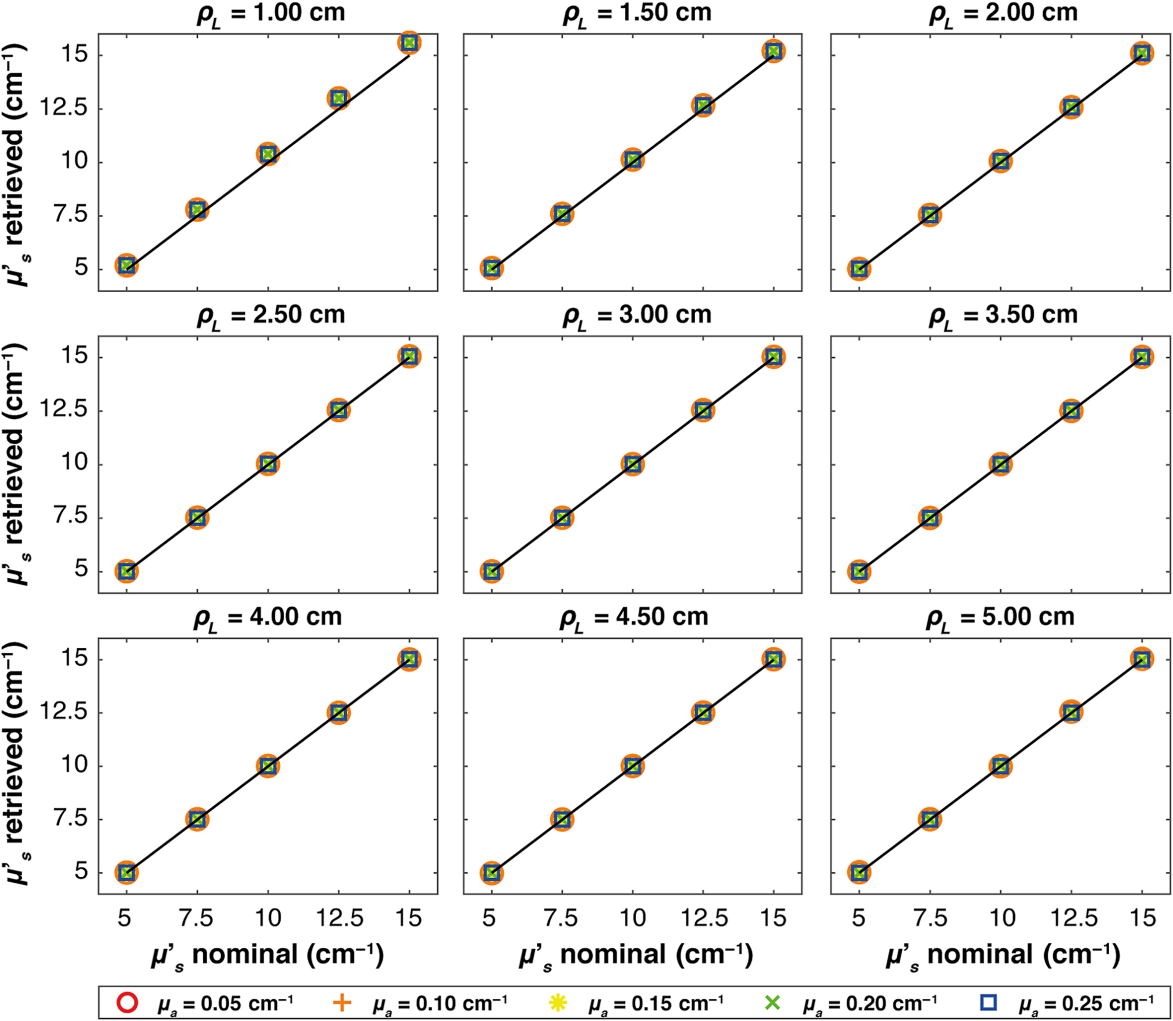
Average retrieved $\mu_{s}^{\prime }$ versus nominal values for each absorption coefficient, represented by different colors and markers across various $\rho_{L}$. Thin solid lines indicate the expected values in each plot. The results are obtained in a homogeneous medium with a Dirac δ IRF.

Figure 2 shows, for the ideal case, the $\mu_{s}^{\prime }$ obtained using Eq. (4) at varying times (evaluated starting from the peak of the IRF) for different $\rho_{L}$. Only data associated to $\mu_{a}=0.15\;{\mathrm{cm}}^{-1}$ are shown. Different colors represent the simulated nominal $\mu_{s}^{\prime }$ values. For all source–detector distances and all values of the nominal reduced scattering coefficient, the estimated $\mu_{s}^{\prime }$ is rather constant as a function of time-of-flight, as expected from Eq. (3). A noisy behavior appears only at time-of-flight corresponding to a very low number of detected photons (either because the time-of-flight is too short for photon to arrive at the detector, or because it is too long and very few photons are detected). By averaging the retrieved $\mu_{s}^{\prime }$ over a wide time-window between 90% of the peak on the rising edge and 10% of the peak on the trailing edge of the curve (represented by colored areas in Fig. 2), a more robust estimate is obtained and reported in Fig. 3 plotted against the corresponding nominal values for all the absorption coefficients (colors and marker) at each $\rho_{L}$. In all plots, the solid line represents the expected values. It is possible to see that for all the cases, the retrieved $\mu_{s}^{\prime }$ corresponds to the nominal one, and indeed, all the plots show linear behaviors. For $\rho_{L}=1\;\mathrm{cm}$, otherwise, there is a small overestimation of the retrieved $\mu_{s}^{\prime }$, with an average error of ∼4%.

#### 4.1.2 Real IRF

**Fig. 4:**
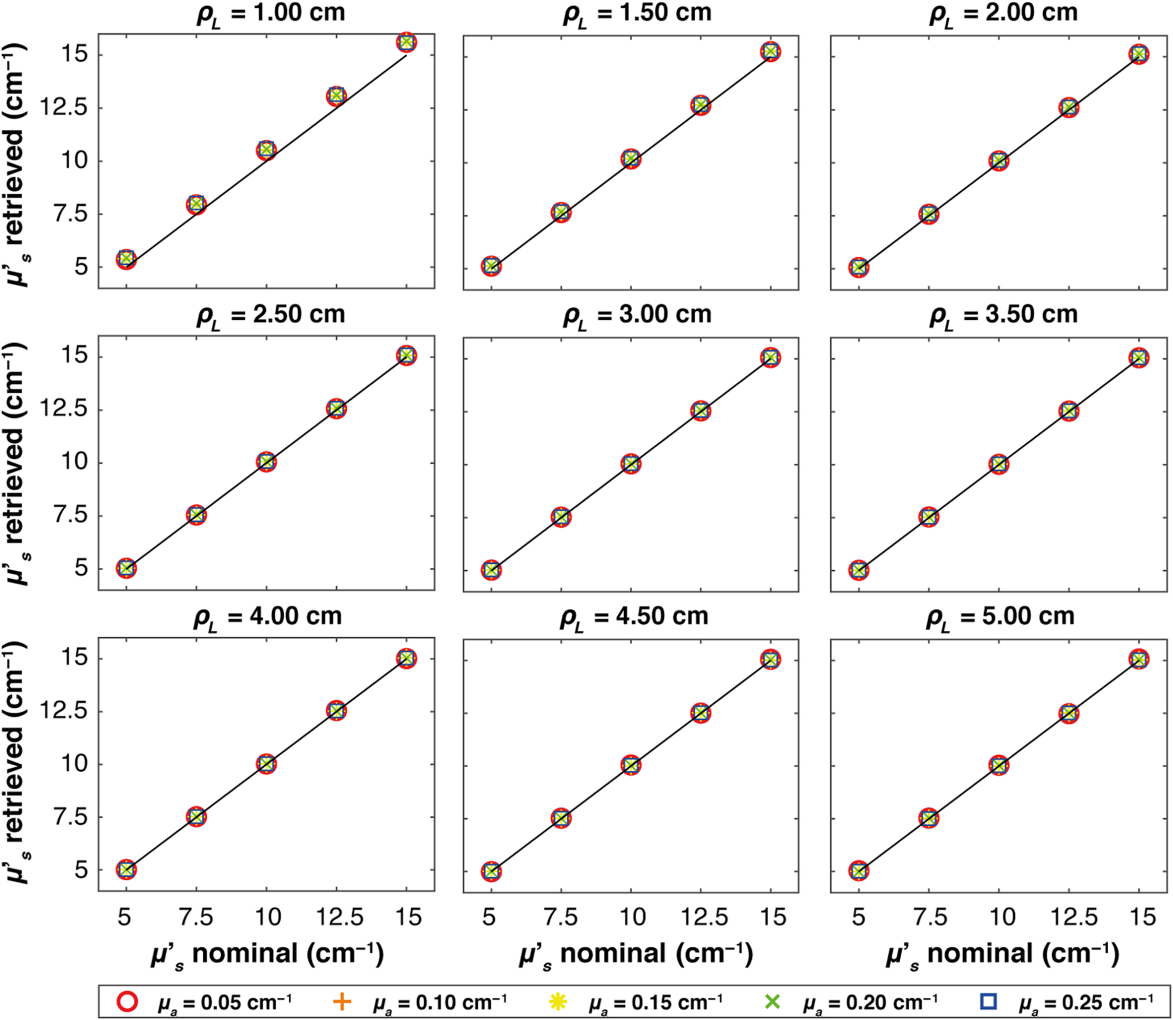
Figure-of-merit values illustrating the average $\mu_{s}^{\prime }$ retrieved versus nominal values for each absorption coefficient, represented by different colors and markers across various $\rho_{L}$. Thin solid lines indicate the expected values in each plot. The results obtained in a homogeneous medium with SNSPD IRF.

**Fig. 5:**
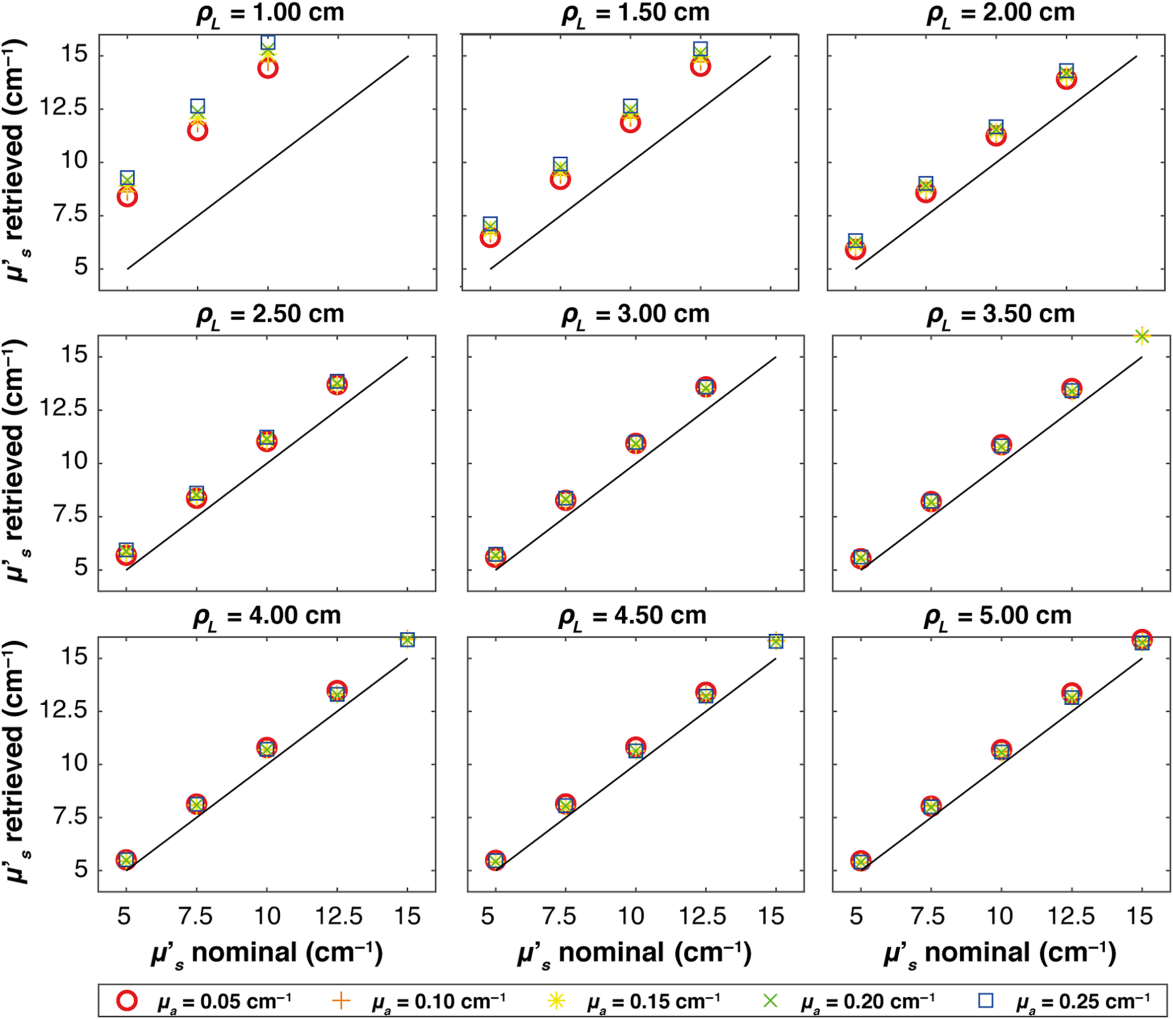
Figure-of-merit values illustrating the average $\mu_{s}^{\prime }$ retrieved versus nominal values for each absorption coefficient, represented by different colors and markers across various $\rho_{L}$. Thin solid lines indicate the expected values in each plot. The results obtained in a homogeneous medium with HYPM IRF.

**Fig. 6:**
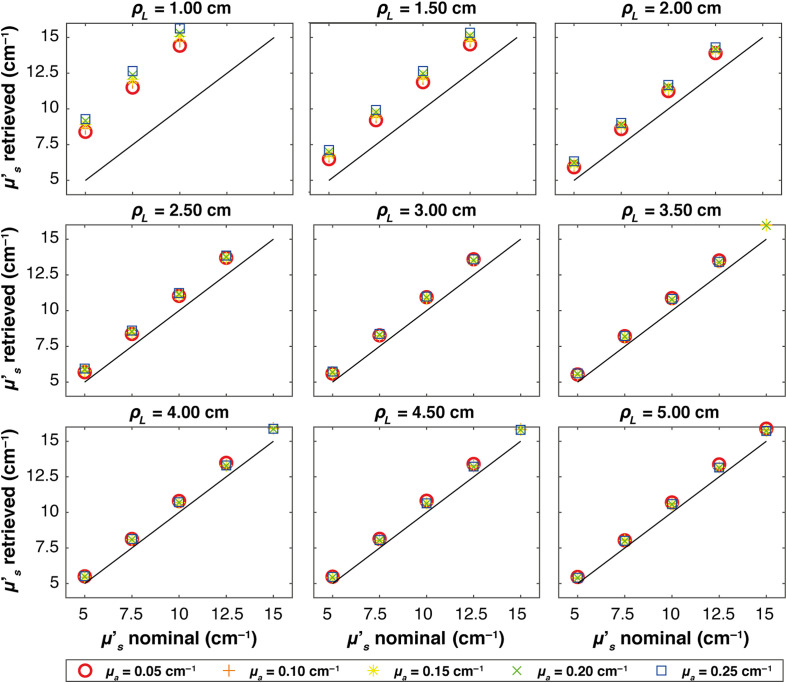
Figure-of-merit values illustrating the average $\mu_{s}^{\prime }$ retrieved versus nominal values for each absorption coefficient, represented by different colors and markers across various $\rho_{L}$. Thin solid lines indicate the expected values in each plot. The results obtained in a homogeneous medium with SiPM IRF.

Figures 4–6 show, at each source–detector distance $\rho_{L}$, the retrieved $\mu_{s}^{\prime }$ values plotted against the nominal values for all absorption coefficients when real systems based on SNSPD, HYPM, and SiPM detectors are used, respectively. For the SNSPD-based system, the results indicate that, similar to the Dirac δ case, the retrieved values are close to the expected ones (i.e., all the points coincide with the expected values line). However, at $\rho_{L}=1\;\mathrm{cm}$, a general overestimation of the computed optical properties is observed (average error of about 6%), even though all points overlap, suggesting no distinction in retrieving $\mu_{s}^{\prime }$ among different values of the absorption coefficients.

By contrast, real systems based on HYPM and SiPM detectors generally exhibit an overestimation of the retrieved scattering coefficient for $\rho_{L}$ smaller than 3.5 cm (e.g., a maximum error of 110% is present at $\rho_{L}=1\;\mathrm{cm}$). At longer distances ($\rho_{L}=4$, 4.5, and 5 cm), the error is smaller: in particular, we observed average error values of 8% for the HYPM-based system and of 7% for the SiPM-based are present. Furthermore, HYPM data show greater variability in the retrieved $\mu_{s}^{\prime }$ depending on the nominal values of the absorption coefficient. A detailed quantification of the estimation errors is reported in Table S1 in the Supplementary Material.

In S2 in the Supplementary Material, we report the relative error across the reconstructed values already shown in Figs. 3–6.

### 4.2 Two-Layer Medium

#### 4.2.1 Ideal IRF (Dirac δ)

**Fig. 7:**
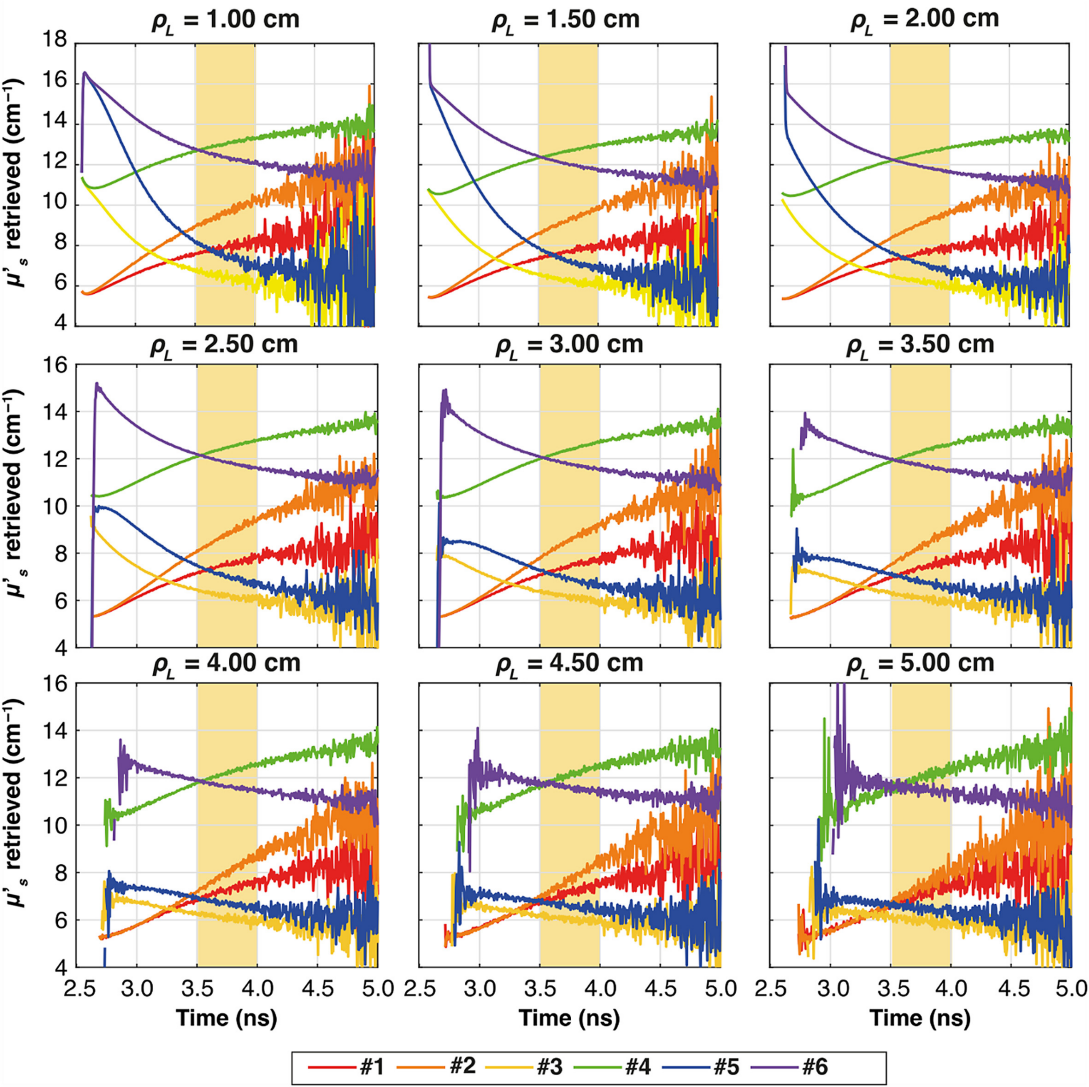
Plots of the retrieved $\mu_{s}^{\prime }$ as a function of time for all the $\rho_{L}$ at all six combinations of two-layer media (colors), for thickness $s_{1}=0.5\;\mathrm{cm}$. In yellow, it is highlighted the time interval used for the calculation of the slope. The results obtained with Dirac δ IRF.

Figure 7 shows the temporal evolution of the estimated $\mu_{s}^{\prime }$ for all the combinations of two-layer media (colors), as listed in Table 1. The results for each $\rho_{L}$ and for the thickness $s_{1}=0.5\;\mathrm{cm}$ and $s_{2}=8.5\;\mathrm{cm}$ are plotted. Furthermore, the region used for the computation of the slope from 3.5 to 4.0 ns is highlighted in yellow.

**Fig. 8:**
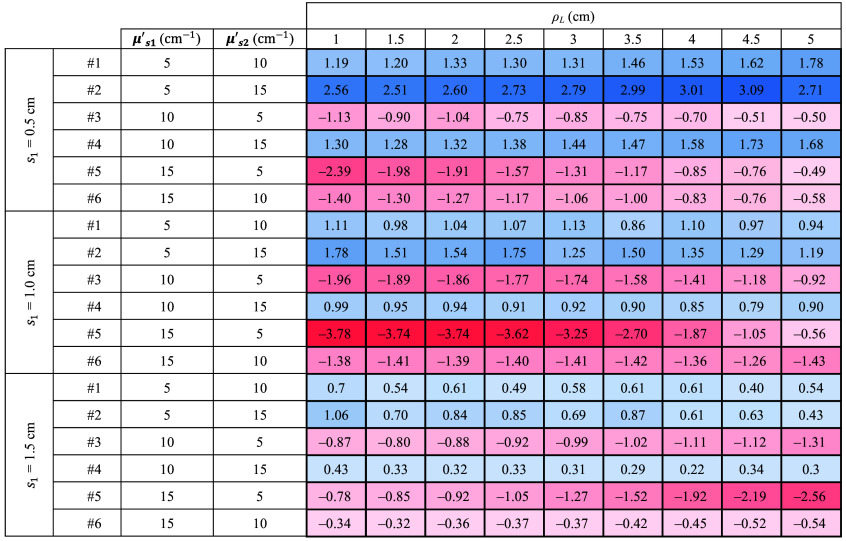
Slope of retrieved $\mu_{s}^{\prime }$ as a function of time for all the $\rho_{L}$ and thicknesses. The results obtained with Dirac δ IRF. For positive (negative) slope values, the cells are colored in blue (red). The intensity of the color depends on the magnitude.

The complete results are listed in Fig. 8, where the slope values are reported for all the sample thicknesses and for each combination of $\mu_{s1}^{\prime }$ and $\mu_{s2}^{\prime }$. For a more intuitive understanding, the color of each cell in the table represents the magnitude of the slope. Specifically, for positive (negative) slope values, the cells are colored in blue (red). The intensity of the color depends on the magnitude.

It is possible to see that in all instances where $\mu_{s1}^{\prime }>\mu_{s2}^{\prime }$, the retrieved $\mu_{s}^{\prime }$ decreases (i.e., the slope is negative and red). Conversely, in cases where $\mu_{s1}^{\prime }<\mu_{s2}^{\prime }$, the slope increases (i.e., it is positive and blue). Moreover, the values are consistent as a greater difference corresponds to a proportional increase in the observed slope. Further results for cases with smaller differences between $\mu_{s1}^{\prime }$ and $\mu_{s2}^{\prime }$ are available in the Supplementary Material, confirming the observed trends and supporting the robustness of the proposed method.

#### 4.2.2 Real IRF

**Fig. 9:**
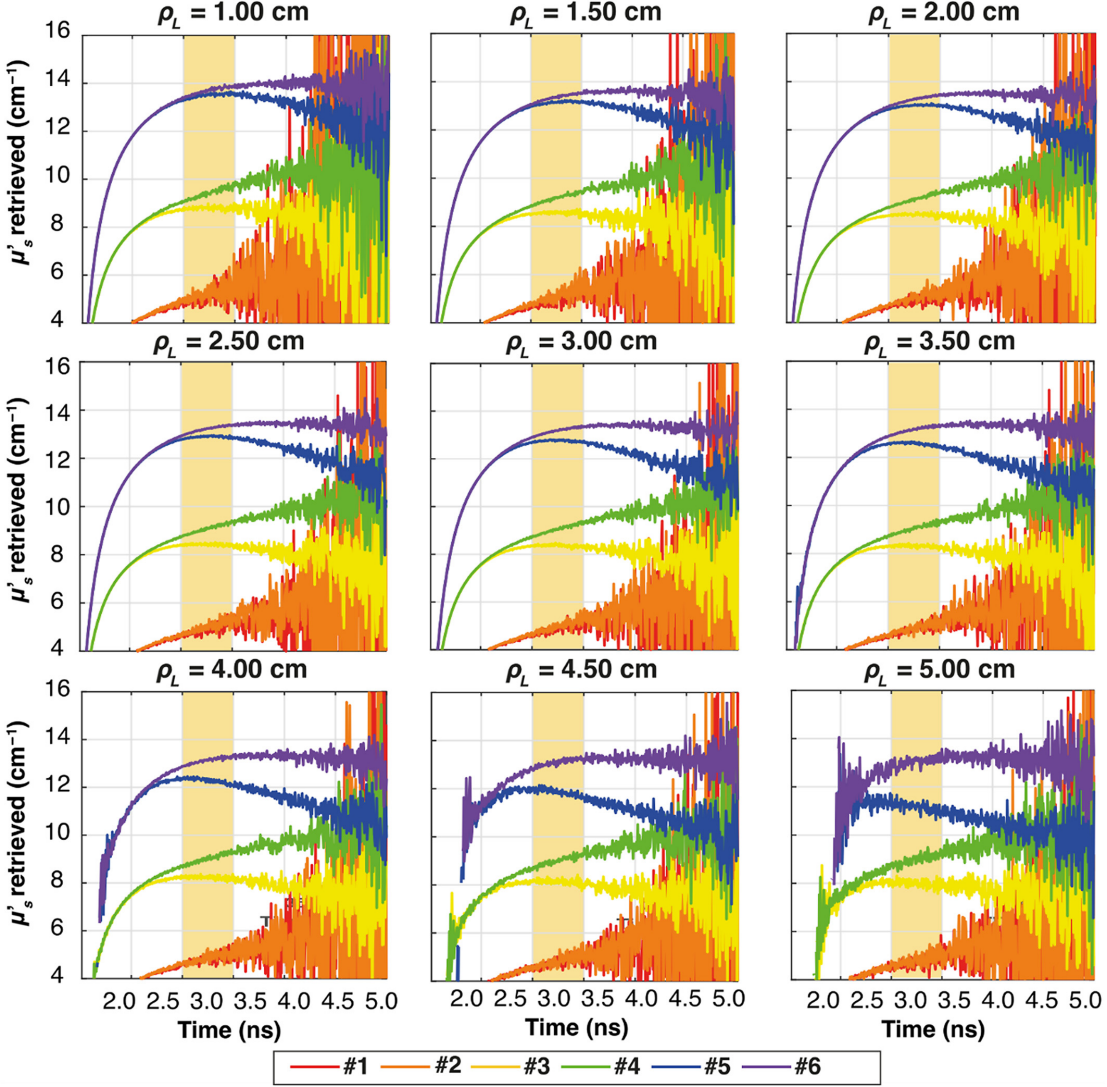
Plots of the retrieved $\mu_{s}^{\prime }$ as a function of time for all the $\rho_{L}$ at all six combinations of two-layer media (colors), for thickness $s_{1}=0.5\;\mathrm{cm}$. In yellow, it is highlighted the time interval used for the calculation of the slope. The results associated with SNSPD IRF.

**Fig. 10:**
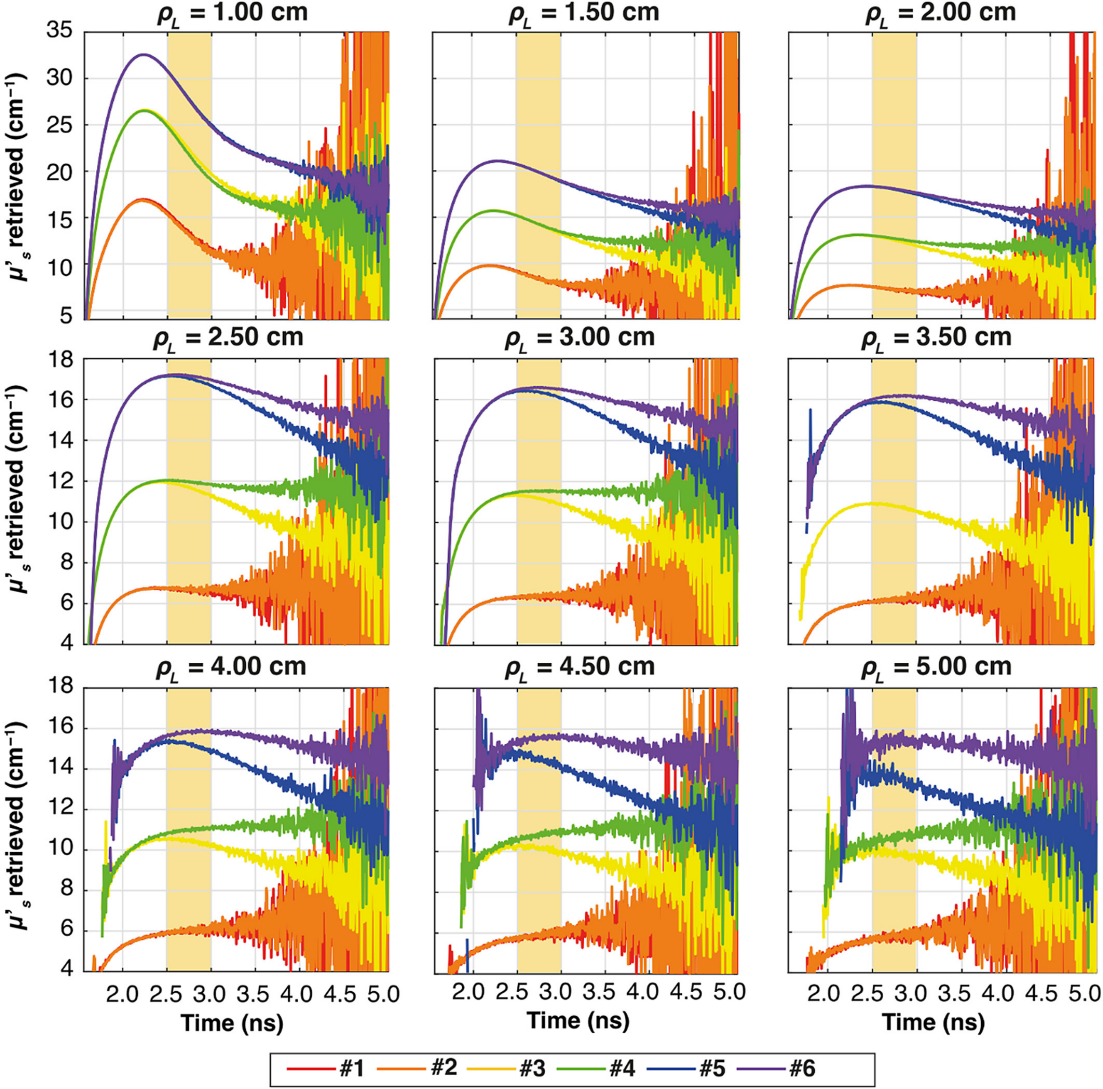
Plots of the retrieved $\mu_{s}^{\prime }$ as a function of time for all the $\rho_{L}$ at all the six combinations of two-layer media (colors), for thickness $s_{1}=0.5\;\mathrm{cm}$. In yellow, it is highlighted the time interval used for the calculation of the slope. The results associated with HYPM IRF.

**Fig. 11:**
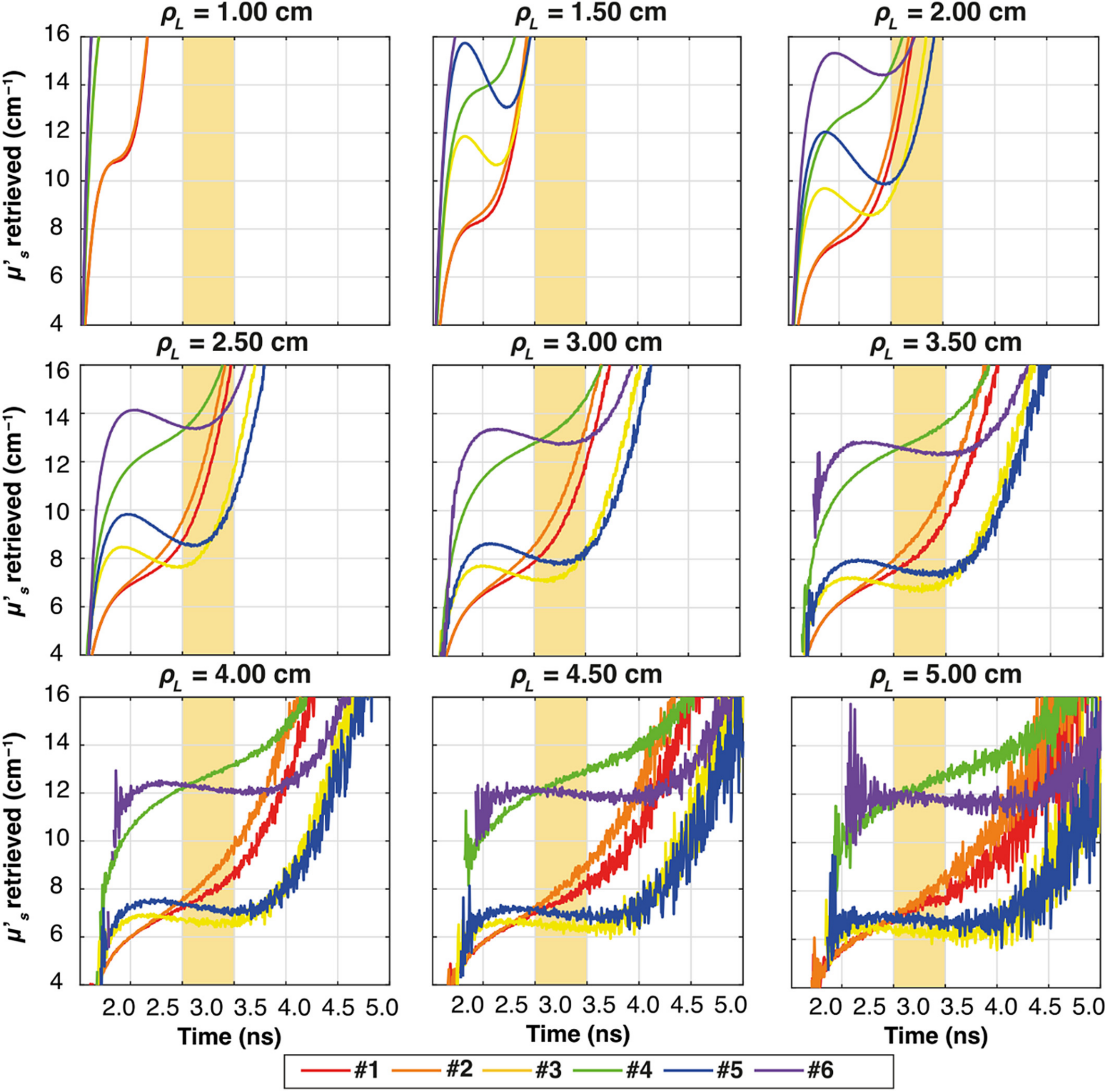
Plots of the retrieved $\mu_{s}^{\prime }$ as a function of time for all the $\rho_{L}$ at all the six combinations of two-layer media (colors), for thickness $s_{1}=0.5\;\mathrm{cm}$. In yellow, it is highlighted the time interval used for the calculation of the slope. The results associated with SiPM IRF.

**Fig. 12:**
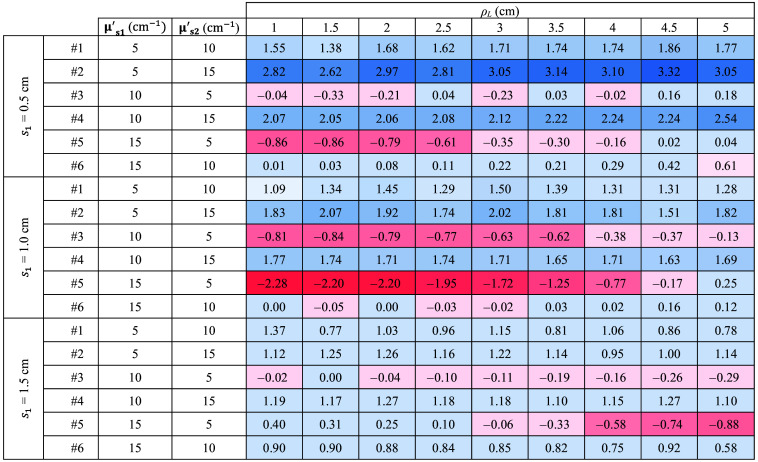
Slope of retrieved $\mu_{s}^{\prime }$ as a function of time for all the $\rho_{L}$ and thicknesses. The results associated with SNSPD IRF. Color code same as in Fig. 11.

**Fig. 13:**
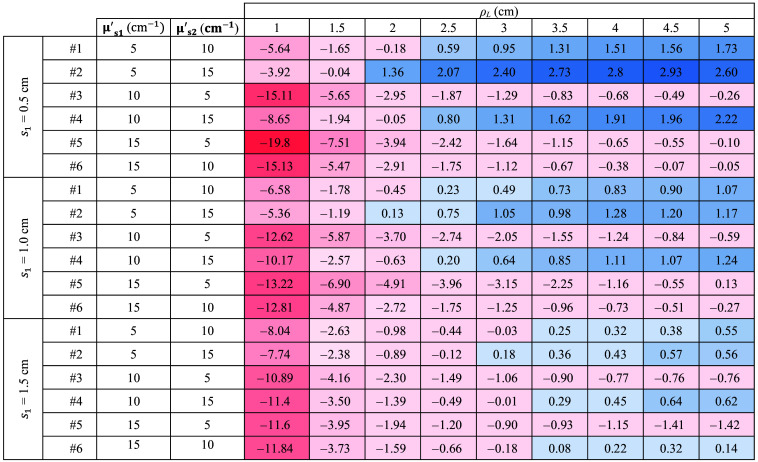
Slope of retrieved $\mu_{s}^{\prime }$ as a function of time for all the $\rho_{L}$ and thicknesses. The results associated with HYPM IRF. Color code same as in Fig. 11.

**Fig. 14:**
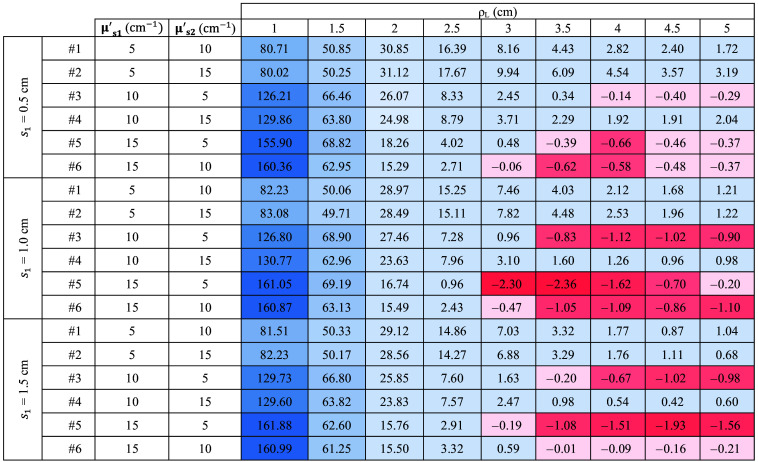
Slope of retrieved $\mu_{s}^{\prime }$ as a function of time for all the $\rho_{L}$ and thicknesses. The results associated with SiPM IRF. Color code same as in Fig. 11.

Similar to Fig. 7 for the ideal system, Figs. 9–11 illustrate the temporal evolution of the estimated $\mu_{s}^{\prime }$ values for all the combinations of two-layer configuration associated with the real systems based on SNSPD, HYPM, and SiPM, respectively. These figures present the results for each $\rho_{L}$ focusing exclusively on thickness $s_{1}=0.5\;\mathrm{cm}$. In addition, the region used to compute the slope is highlighted in yellow. The complete corresponding results are provided in Figs. 12–14, categorized by IRF. The same methodology described in the previous section was applied to color the table cells.

The highlighted region starts at different times because the position of the IRF peak varies on the system (Dirac δ = 2.5 ns, SIPM = 2.0 ns, HYPM = 1.6 ns, SNSPD = 2.0 ns). We have in fact not aligned the time position of the different IRFs. Despite these differences, all regions are consistently set to begin 1 ns after the IRF peak. This approach was carefully chosen to ensure that the analysis focuses on the relevant portion of the curve. By starting 1 ns after the peak, we can confidently examine the part of the curve where the slope can be associated with changes in the reduced scattering coefficient.

The results associated with the SNSPD (Fig. 9) show that most of the $\rho_{L}$ exhibit curves from which the slope can be extracted. However, for sample #6, the curve displays a flat behavior, resulting in a slope that is close to zero (see Fig. 12).

As for the homogeneous case, both HYPM and SiPM provide less meaningful information for short $\rho_{L}$ values. In the case of HYPM, the estimated slope values provide information on the direction of change in the reduced scattering coefficients of the two layers only for $\rho_{L}$ greater than 2.5, 3.5, and 4.5 cm for thicknesses $s_{1}=0.5$, 1.0, and 1.5 cm, respectively (see Fig. 13). By contrast, when using the SiPM IRF, only $\rho_{L}\geq 4.0\;\mathrm{cm}$ can be used (see Fig. 14). Notably, there is no substantial difference across the various thicknesses analyzed.

However, the same saturating behavior observed in sample #6 persists, limiting the interpretability of these results for the last two IRFs.

It is important to highlight that for all the cases studied here, the absolute values of the reconstructed scattering for the two layers do not match the nominal values of either of the two layers. This discrepancy points to limitations in the reconstruction process presented in this study.

## 5 Discussion and Conclusion

We have explored the possibility of using the SRS approach in the TD. From a simple theoretical derivation, we have shown that in a homogeneous sample, the reduced scattering coefficient could be derived from the spatial derivative of the attenuation. We have verified this by means of numerical simulations at different source–detector distances, including also the effect of realistic IRFs. The retrieved $\mu_{s}^{\prime }$ is characterized by acceptable errors (less than 10%), apart from the case of using a short source–detector distance, as expected from the theoretical derivation and the numerical implementation of the adopted method.

We have also tested the case of a two-layer medium, a more realistic configuration that better mimics human tissues such as the skeletal muscle covered by an adipose tissue layer or the cerebral cortex enclosed in the extracerebral tissues (i.e., scalp, skull, and cerebrospinal fluid). By observing the results of simulations in the two-layer medium, we noted a dependence on time of the retrieved $\mu_{s}^{\prime }$. This dependence can be expected and is due to the presence of different $\mu_{s}^{\prime }$ values. Then, we decided to use a linear fit as a first-order method to give an estimation of this trend, to evaluate the heterogeneity of scattering properties in the medium. Surely, this is an oversimplifying assumption, that, however, has the advantage of simplicity.

As for the influence of the absorption coefficient $\mu_{a}$, we have simulated different values for $\mu_{a}$ in a range that is typical for biological tissues (0.05 to $0.25\;{\mathrm{cm}}^{-1}$). In the case of a Dirac δ IRF, as the only dependence on $\mu_{a}$ of the solution of the photon diffusion equation is in the factor $e^{-\mu_{a}vt}$, as can be seen in Eq. (1), the dependence on $\mu_{a}$ in the derivative of the attenuation disappears [see Eq. (2)]. In the case of other IRFs, instead, some dependence from $\mu_{a}$ is present in the $\mu_{s}^{\prime }$ estimation, due to the convolution operation. Nevertheless, we would expect a major effect of $\mu_{a}$ in a reduction in the number of detected photons, with limited effects on the spatial gradient of the attenuation.

Not surprisingly, the TD SRS approach, being based on a homogeneous theory, is not able to recover absolute $\mu_{s}^{\prime }$ values in this case. However, the presence of a change in the reduced scattering coefficient of the two layers can be noted by looking at the temporal trend of the derived $\mu_{s}^{\prime }$. This information can be of interest for improving the data analysis procedures based on the use of physical models for photon migration in a two-layer geometry. Keeping $\mu_{s}^{\prime }$ constant in the two layers, indeed, might introduce errors in the estimate of the absorption coefficient, whereas the information on spatial changes of $\mu_{s}^{\prime }$ could be beneficial.

The simulations have also shown that the temporal characteristics of the IRF are crucial for obtaining reliable results. Specifically, the SNSPD detector characterized by the best IRF in terms of narrow response without afterpulsing shows the best performance. However, nowadays, such a detector comes only with a very narrow area (diameter <10  μm) that strongly limits the use in diffuse optics laboratory and *in vivo* settings. On the other hand, the HYPM and the SiPM offer better solutions in terms of sensitivity thanks to wider area but are limited by broader response (and longer tail): this prevents their use for TD SRS at source–detector distances shorter than ∼3  cm, unavoidably reducing the overall signal-to-noise.

We have studied the efficacy of the TD SRS approach in the two-layer medium that closely resembles the real case of NIRS measurements on muscle covered by an adipose tissue layer. The two-layer model can also mimic, to a first approximation, the more complex case of NIRS measurements on the head, if its structure is described by the extra- and intra-cerebral macro compartments. For more complex, multilayered geometries, we expect that the TD SRS method (with a single pair of source detector distances) would produce worse results compared with the two-layer medium. The use of the TD SRS method at multiple source detector distances (e.g., narrow and large distances to better sample superficial and deeper layers) could potentially improve the results. By combining TD SRS measurements from multiple source–detector distances would add additional data that could be used for improving spatial resolution like it is typically done in fNIRS measurements with the high-density approach.^17^

In conclusion, the TD SRS approach could be used for a simple estimate of the reduced scattering coefficient in a homogeneous sample. In a future work, this method will be applied to a more realistic set of numerical simulations obtained by means of Monte Carlo tools, to assess possible improvements in the reduced scattering coefficient estimation with respect to a classical TD measurement at a single source–detector distance and the use of a nonlinear fitting procedure.^18^ As for a two-layer sample, the TD SRS approach can be a simple way to estimate spatial changes of $\mu_{s}^{\prime }$ but not the absolute value of $\mu_{s}^{\prime }$. Finally, care should be taken to use a TD system with proper IRF.

## Supplementary Material

10.1117/1.JBO.30.7.075002.s01

## Data Availability

The codes and datasets generated during and analyzed during the current study are available from the corresponding author upon reasonable request.
