# Supplementary material for: Evaluation of layered tissue scattering properties: a time-domain spatially resolved spectroscopy approach
Source: J Biomed Opt. 2025 Jul 21;30(7):075002. doi: 10.1117/1.JBO.30.7.075002 (PMC12279310; doi:10.1117/1.JBO.30.7.075002)
Supplement: Supplementary file 1 [file JBO_030_075002_SD001.pdf]

Supplementary Material:

Evaluation of Layered Tissue Scattering Properties: A Time-Domain Spatially Resolved Spectroscopy Approach (TD SRS)

Elisabetta Avanzi,[a] Laura Di Sieno,[a] Alberto Dalla Mora,[a] Lorenzo Spinelli,[b] Alessandro Torricelli[a,b]*

[a]Dipartimento di Fisica, Politecnico di Milano, Milan, Italy.

[b]Istituto di Fotonica e Nanotecnologie, Consiglio Nazionale delle Ricerche, Milan, Italy

S1. Additional cases in the two-layer medium configuration

We provide here additional simulations aimed at further clarifying the behavior of the slope-based method when the reduced scattering coefficients in the two-layer model are very similar. To address this, we performed new simulations limited to the case of the ideal impulse response function (i.e., Dirac δ IRF). In addition to the previously reported cases (see Table 1 of the main manuscript), where the difference between μ'_{s1} values was relatively large, we now consider a series of new configurations in which μ'_{s1} and μ'_{s2} differ only slightly. These cases, presented in Fig S.1-3, span differences ($\Delta\mu'_{s12}$) from 1 to 4 cm^{-1}, both in increasing and decreasing order.

The slopes obtained for different ρ_L and three values of superficial layer thickness are reported in Table S1. From these results, we observe that when the difference between μ'_{s1} and μ'_{s2} is minimal ($\Delta\mu'_{s12}$ = 1 cm^{-1}, cases #7 and #11), the slope remains close to zero across all distances and thicknesses, suggesting limited sensitivity to small inhomogeneities in scattering. As $\Delta\mu'_{s12}$ increases, the slope values become progressively more pronounced, indicating a growing sensitivity to mismatch between the two layers.

The effect of the superficial layer thickness on the slope behavior can also be evaluated, by comparing results across three different thicknesses. For small differences between the scattering coefficients of the two layers the slope remains close to zero across all thicknesses and source-detector distances. In this regime, there is no clear dependence on s_1, suggesting that the method is largely insensitive both to small $\Delta\mu'_{s12}$ values and the superficial layer thickness when the tissue is nearly homogeneous. As the difference in scattering increases a clearer trend emerges: the slope values tend to be larger (in absolute terms) when the superficial

layer is thinner. For instance, in case #8, the slopes observed with s_1 = 0.5 cm are consistently higher than those obtained with s_1 = 1.5 cm. In cases with large differences in scattering (cases #10 and #14) the effect of thickness remains evident. The magnitude of the slope stays high for all values of s_1, but again, the largest values tend to occur with the smallest s_1. Notably, for case #14, the negative slope becomes increasingly pronounced as the thickness decreases. Overall, thinner superficial layers lead to stronger slope variations and improved detectability of heterogeneities in deeper layers, particularly when the difference in μ'_s between the layers is moderate or large.

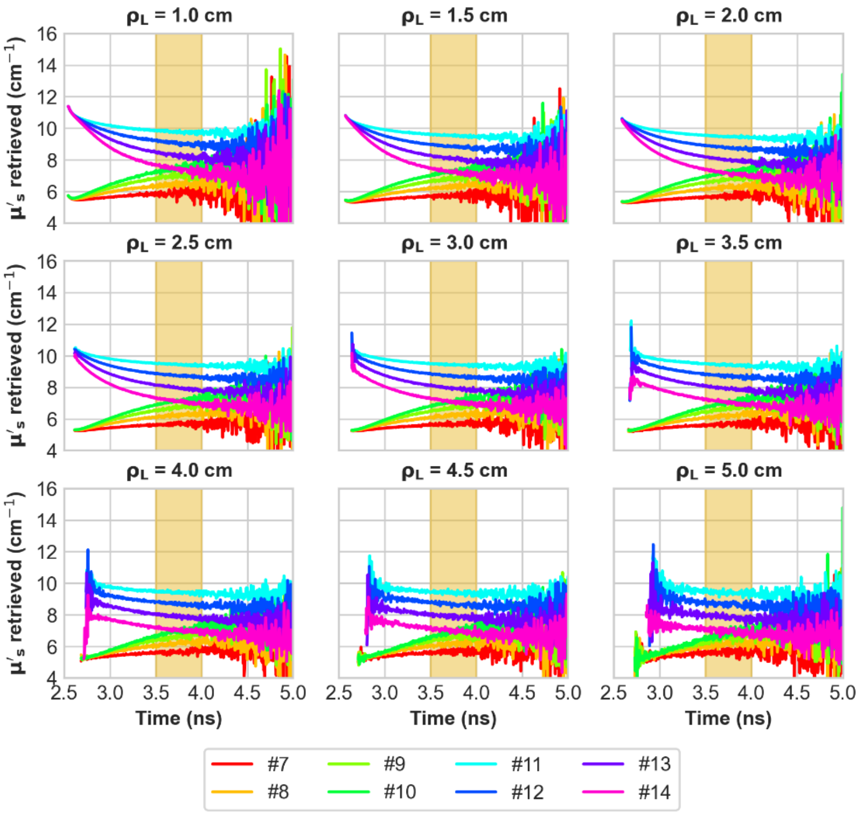

Fig. S1 Plots of the retrieved μ'_s as a function of time for all the ρ_L at all the 8 combinations of two-layer media (colors), for thickness s_1 = 0.5 cm. In yellow it is highlighted the time interval used for the calculation of the slope. Results associated to Dirac δ IRF.

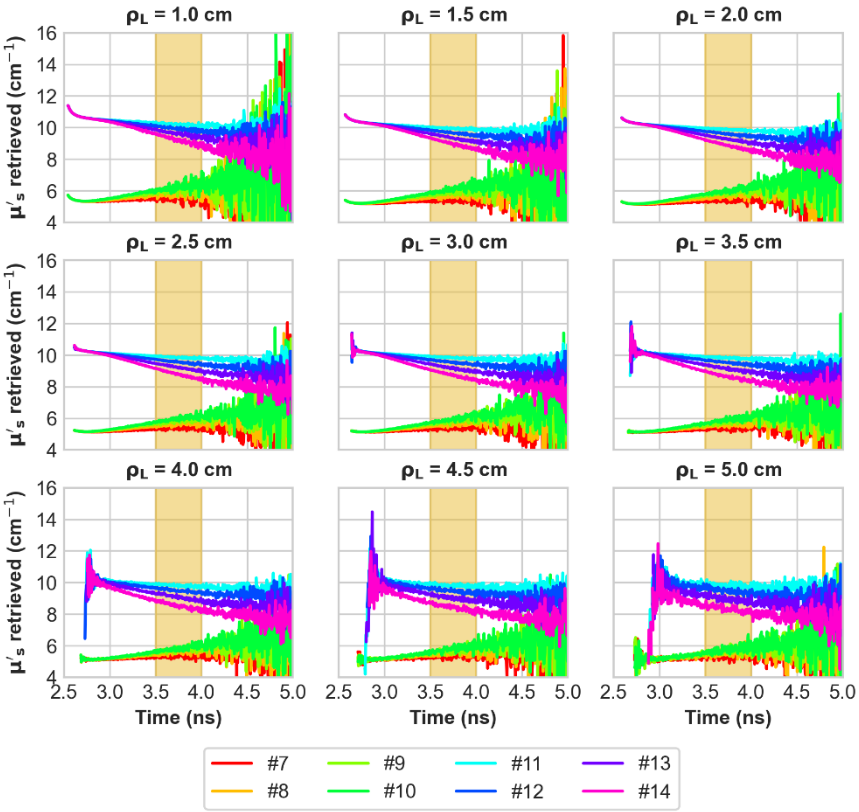

Fig. S2 Plots of the retrieved μ'_s as a function of time for all the ρ_L at all the 8 combinations of two-layer media (colors), for thickness s_1 = 1.0 cm. In yellow it is highlighted the time interval used for the calculation of the slope. Results associated to Dirac δ IRF.

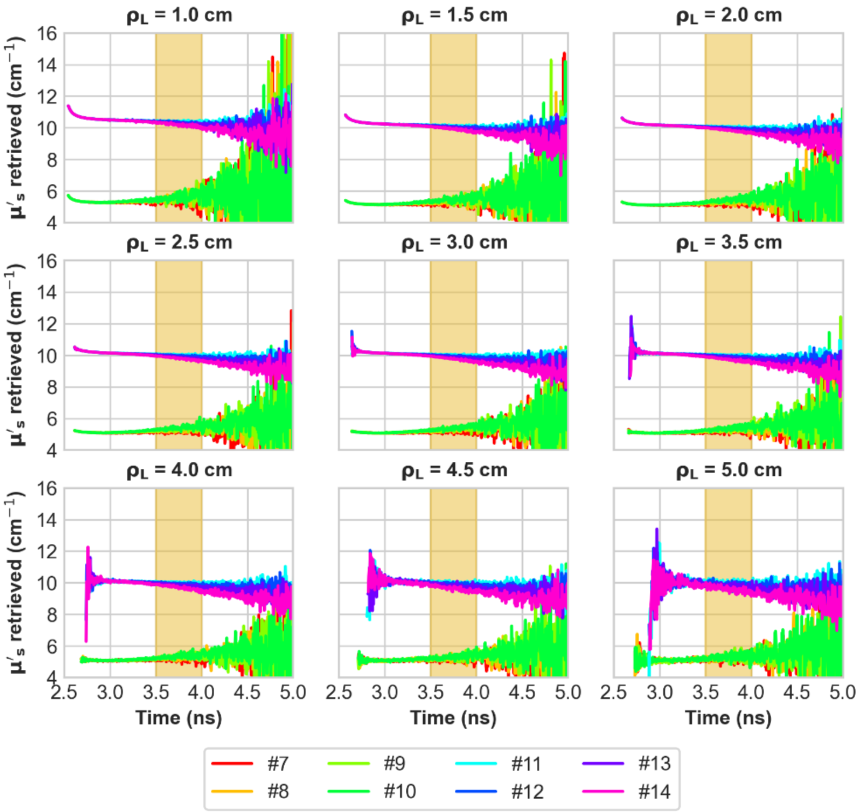

Fig. S3 Plots of the retrieved μ'_s as a function of time for all the ρ_L at all the 8 combinations of two-layer media (colors), for thickness s_1 = 1.5 cm. In yellow it is highlighted the time interval used for the calculation of the slope. Results associated to Dirac δ IRF.

Table S1. Slope of retrieved μ'_s for all the ρ_L and thicknesses. Results obtained with Dirac δ IRF. For positive (negative) slope values the cells are colored in blue (red). The intensity of the color depends on the magnitude.

				ρ_L (cm)								
		μ'_{s1} (cm^{-1})	μ'_{s2} (cm^{-1})	1	1.5	2	2.5	3	3.5	4	4.5	5
s_1 = 0.5 cm	#7	5	6	0.18	0.25	0.27	0.08	0.22	0.20	0.24	0.15	0.13
	#8	5	7	0.42	0.58	0.39	0.41	0.43	0.52	0.48	0.68	0.27
	#9	5	8	0.43	0.69	0.79	0.73	0.70	0.82	0.82	0.75	1.11
	#10	5	9	0.95	0.97	1.01	0.91	1.16	1.02	1.16	1.33	1.33
	#11	10	9	-0.28	-0.29	-0.23	-0.30	-0.24	-0.25	-0.22	-0.19	-0.31
	#12	10	8	-0.56	-0.47	-0.50	-0.46	-0.47	-0.44	-0.45	-0.38	-0.36
	#13	10	7	-0.68	-0.75	-0.61	-0.69	-0.64	-0.51	-0.53	-0.46	-0.58
	#14	10	6	-0.96	-0.83	-0.80	-0.76	-0.74	-0.76	-0.52	-0.57	-0.56
s_1 = 1.0 cm	#7	5	6	0.25	0.23	0.20	0.31	0.25	0.31	0.18	0.31	0.30
	#8	5	7	0.63	0.40	0.52	0.41	0.50	0.41	0.58	0.44	0.50
	#9	5	8	0.59	0.65	0.65	0.74	0.62	0.85	0.59	0.79	0.76
	#10	5	9	0.71	0.97	0.84	0.78	1.03	0.78	0.86	0.91	0.93
	#11	10	9	-0.35	-0.29	-0.32	-0.27	-0.30	-0.30	-0.35	-0.24	-0.28
	#12	10	8	-0.66	-0.66	-0.61	-0.62	-0.61	-0.59	-0.59	-0.67	-0.70
	#13	10	7	-0.99	-1.03	-0.98	-0.96	-0.99	-0.86	-0.98	-0.84	-0.78
	#14	10	6	-1.46	-1.40	-1.39	-1.38	-1.29	-1.23	-1.19	-1.12	-0.83
s_1 = 1.5 cm	#7	5	6	0.00	0.22	0.31	0.10	0.15	0.17	0.23	0.09	0.38
	#8	5	7	0.70	0.10	0.42	0.25	0.45	0.23	0.28	0.34	0.31
	#9	5	8	0.73	0.37	0.50	0.40	0.46	0.33	0.37	0.42	0.39
	#10	5	9	0.72	0.45	0.49	0.55	0.55	0.49	0.45	0.54	0.26
	#11	10	9	-0.14	-0.14	-0.17	-0.15	-0.13	-0.13	-0.15	-0.21	-0.21
	#12	10	8	-0.32	-0.25	-0.30	-0.29	-0.29	-0.30	-0.29	-0.32	-0.14
	#13	10	7	-0.41	-0.45	-0.42	-0.49	-0.49	-0.48	-0.44	-0.56	-0.40
	#14	10	6	-0.58	-0.67	-0.65	-0.63	-0.71	-0.69	-0.81	-0.83	-0.87

S2. Homogeneous medium: influence of μ_a on μ'_s

To complement the analysis presented in Fig. 3-6 of the main manuscript a quantitative evaluation of the estimation accuracy of μ'_s was performed by computing the relative percentage error across the reconstructed values already shown in those figures. The goal was to assess how the absorption coefficient influences the performance of μ'_s estimation for different IRFs.

Table S2 summarizes the computed relative percentage error ε for μ'_s as a function of μ_a and ρ_L for each IRF. As expected, the Dirac δ and SNSPD IRFs result in a very low error (ε <1.5%) regardless of the absorption level for all $\rho_L \geq 2$ cm, and a slightly larger error (ε <6.2%) at shorter ρ_L, indicating that sharp or near-ideal temporal responses allow the algorithm to remain largely unaffected by absorption changes. In contrast, broader IRFs such as HYPM and SiPM show a clear trend of increasing error with higher μ_a and decreasing ρ_L. The error is larger than 20% for $\rho_L < 2.5$ cm and for $\rho_L < 2.0$ cm, respectively for HYPM and SiPM. Although the errors remain within a moderate range, these findings highlight a growing sensitivity to absorption when the temporal resolution of the system is limited.

Overall, this analysis confirms that the impact of absorption on μ'_s estimation accuracy is strongly modulated by the IRF of the system.

Table S2 Relative percentage error ε for μ'_s as a function of μ_a and ρ_L for each IRF. Color code: white ε <5%, green 5%≤ ε < 10%, yellow 10% ≤ ε < 20 %, red 20% ≤ ε < 40%, dark red ε ≥ 40%.

		ρ_L (cm)								
	μ_a (cm^{-1})	1	1.5	2	2.5	3	3.5	4	4.5	5
Dirac δ IRF	0.05	4.0	1.4	0.7	0.4	0.3	0.2	0.2	0.1	0.1
	0.10	4.0	1.5	0.7	0.4	0.3	0.2	0.1	0.1	0.1
	0.15	4.1	1.3	0.7	0.4	0.2	0.3	0.1	0.2	0.1
	0.20	3.9	1.5	0.7	0.4	0.2	0.3	0.2	0.1	0.1
	0.25	3.8	1.3	0.8	0.4	0.2	0.2	0.1	0.2	0.0
SNSPD	0.05	5.3	1.9	1.0	0.6	0.4	0.3	0.3	0.3	0.4
	0.10	5.5	2.1	1.1	0.6	0.4	0.3	0.3	0.1	0.0
	0.15	5.8	2.2	1.1	0.7	0.5	0.3	0.3	0.2	0.2
	0.20	6.0	2.3	1.2	0.7	0.5	0.4	0.3	0.3	0.2
	0.25	6.2	2.4	1.3	0.8	0.6	0.4	0.3	0.3	0.2
HYPM	0.05	85.6	35.8	19.6	12.6	9.0	6.9	5.6	4.5	4.3
	0.10	93.0	41.3	24.1	16.2	12.0	9.4	7.7	6.4	5.5
	0.15	99.5	46.1	27.9	19.3	14.5	11.5	9.4	8.0	7.1
	0.20	106.1	50.3	31.3	22.0	16.7	13.4	11.1	9.3	8.3
	0.25	111.3	54.2	34.2	24.4	18.7	15.1	12.6	10.7	9.4
SiPM	0.05	47.1	20.4	13.4	10.9	9.8	8.9	8.3	8.1	7.2
	0.10	50.6	22.7	14.3	10.9	9.1	8.0	7.3	6.7	6.4
	0.15	54.0	24.9	15.6	11.5	9.4	8.0	7.2	6.4	6.1
	0.20	57.3	27.0	17.0	12.4	9.9	8.4	7.3	6.6	6.0
	0.25	60.1	28.8	18.3	13.3	10.5	8.8	7.6	6.9	6.1
